# A cross-sectional quantitative analysis of the readability and quality of online resources regarding thumb carpometacarpal joint replacement surgery

**DOI:** 10.1016/j.jham.2024.100119

**Published:** 2024-06-26

**Authors:** Brandon Lim, Suddhajit Sen

**Affiliations:** a Trinity College Dublin, School of Medicine, Dublin, Ireland; b Department of Trauma and Orthopaedic Surgery, Raigmore Hospital, Inverness, UK

**Keywords:** Health literacy, Hand surgery, Thumb surgery, Thumb carpometacarpal joint surgery, Internet

## Abstract

**Background:**

Thumb carpometacarpal (CMC) joint osteoarthritis is a common degenerative condition that affects up to 15 ​% of the population older than 30 years. Poor readability of online health resources has been associated with misinformation, inappropriate care, incorrect self-treatment, worse health outcomes, and increased healthcare resource waste. This study aims to assess the readability and quality of online information regarding thumb carpometacarpal (CMC) joint replacement surgery.

**Methods:**

The terms “thumb joint replacement surgery”, “thumb carpometacarpal joint replacement surgery”, “thumb cmc joint replacement surgery”, “thumb arthroplasty”, “thumb carpometacarpal arthroplasty”, and “thumb cmc arthroplasty” were searched in Google and Bing. Readability was determined using the Flesch Reading Ease Score (FRES) and the Flesch-Kincaid Reading Grade Level (FKGL). FRES >65 or a grade level score of sixth grade and under was considered acceptable. Quality was assessed using the Patient Education Materials Assessment Tool (PEMAT) and a modified DISCERN tool. PEMAT scores below 70 were considered poorly understandable and poorly actionable.

**Results:**

A total of 34 websites underwent qualitative analysis. The average FRES was 54.60 ​± ​7.91 (range 30.30–67.80). Only 3 (8.82 ​%) websites had a FRES score >65. The average FKGL score was 8.19 ​± ​1.80 (range 5.60–12.90). Only 3 (8.82 ​%) websites were written at or below a sixth-grade level. The average PEMAT percentage score for understandability and actionability was 76.82 ​± ​9.43 (range 61.54–93.75) and 36.18 ​± ​24.12 (range 0.00–60.00) respectively. Although 22 (64.71 ​%) of websites met the acceptable standard of 70 ​% for understandability, none of the websites met the acceptable standard of 70 ​% for actionability. The average total DISCERN score was 32.00 ​± ​4.29 (range 24.00–42.00).

**Conclusions:**

Most websites reviewed were written above recommended reading levels. Most showed acceptable understandability but none showed acceptable actionability. To avoid the negative outcomes of poor patient understanding of online resources, providers of these resources should optimise accessibility to the average reader by using simple words, avoiding jargon, and analysing texts with readability software before publishing the materials online. Websites should also utilise visual aids and provide clearer pre-operative and post-operative instructions.

## 1 Introduction

Health literacy is defined as “the ability of an individual to obtain and translate knowledge and information to maintain and improve health in a way that is appropriate to the individual and system contexts”.^1^ Poor health literacy has been associated with greater inpatient hospital service utilization, higher postoperative complications and costs, and lower post-operative rehabilitation compliance.^2^, ^3^, ^4^, ^5^, ^6^, ^7^, ^8^ Meanwhile, poor readability of online health resources is leads to misinformation, inappropriate care, incorrect self-treatment, worse health outcomes, and increased healthcare resource waste.^9^ Studies have shown that the average American reads at a seventh to eighth-grade level while most online healthcare-related materials are written at or above a tenth-grade level.^10^, ^11^, ^12^ Previous studies on the readability of online healthcare-related resources have determined that patient education materials need to be written at or below a US sixth-grade level to be deemed acceptable for the public.^13^, ^14^, ^15^, ^16^

Thumb carpometacarpal (CMC) joint osteoarthritis is a common and painful degenerative condition that affects up to 15 ​% of the population older than 30 years and almost 33 ​% of postmenopausal women.^17^^,^^18^ Surgeries such as trapeziectomy, arthrodesis, or thumb CMC joint replacement can be offered after conservative managements fail.^17^ The first thumb CMC joint replacement was described by de la Caffinière in 1974,^19^ making it a relatively newer treatment for severe thumb CMC joint osteoarthritis compared to the trapeziectomy which was introduced in 1949.^20^ Studies have shown that thumb CMC joint replacements have significant advantages in strength and range of motion compared to trapeziectomies, while also having low incidences of prosthesis instability.^21^, ^22^, ^23^ A literature search found that although there were analyses of the readability and quality of online resources regarding osteoarthritis in general and of pathologies of the hand and wrist, there were no studies assessing the readability of online resources regarding thumb carpometacarpal joint replacement surgery.

This study aims to assess the readability of information available on the internet regarding thumb carpometacarpal joint replacement surgery using readability scoring systems and compare these with previously recommended standards.

## 2 Methods

### 2.1 Search strategy

In June 2024, websites with patient information regarding thumb carpometacarpal joint replacement surgery were identified using Google and Bing, the two largest search engines by market share at the time of this investigation.^24^ Cookies, location, and user account information were disabled before each search to avoid any unintended bias in search results. Search terms were identified using Google Trends. Search terms used ranged from lower complexity terms (“thumb joint replacement surgery” and “thumb arthroplasty”) to higher complexity terms (“thumb carpometacarpal joint replacement surgery”, “thumb cmc joint replacement surgery”, “thumb carpometacarpal arthroplasty”, and “thumb cmc arthroplasty”), resulting in a total of 12 unique searches. Table 1 shows the number of hits returned from each search engine and search term combination.

**Table 1:** Hits returned for each search engine and search term combination.

Search engine	Hits returned
Google & thumb joint replacement surgery	11,100,000
Google & thumb arthroplasty	2,080,000
Google & thumb carpometacarpal joint replacement surgery	416,000
Google & thumb cmc joint replacement surgery	225,000
Google & thumb carpometacarpal joint arthroplasty	111,000
Google & thumb cmc joint arthroplasty	66,800
Bing & thumb joint replacement surgery	847,000
Bing & thumb arthroplasty	1,530,000
Bing & thumb carpometacarpal joint replacement surgery	460,000
Bing & thumb cmc joint replacement surgery	439,000
Bing & thumb carpometacarpal joint arthroplasty	103,000
Bing & thumb cmc joint arthroplasty	109,000

**Fig. 1:**
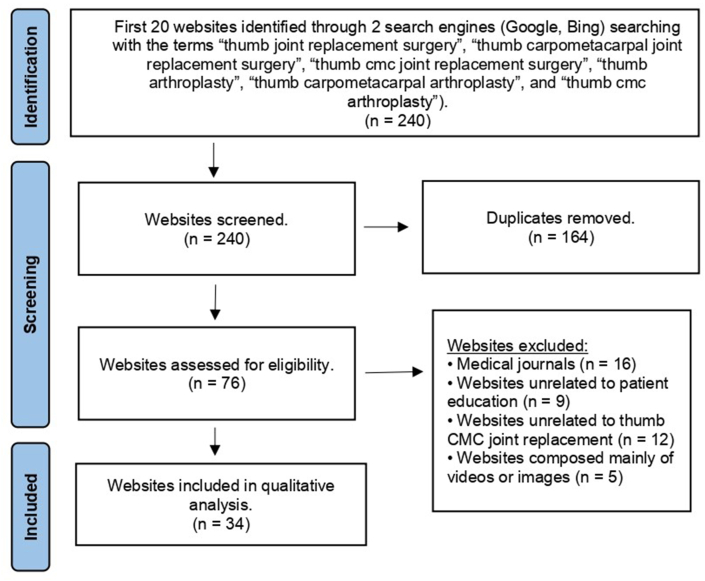
Flow diagram of the methodology used for screening websites (based on the Preferred Reporting Items for Systematic Reviews and Meta-Analyses statement).

The first 20 results from each of the 12 unique searches, a total of 240 websites, were screened. This limitation was set according to search strategies utilized by previous studies that showed that most people do not look beyond the first two pages of results on a search engine.^14^, ^15^, ^16^^,^^25^, ^26^, ^27^, ^28^ Non-functional websites, duplicate websites, websites unrelated to patient information regarding thumb carpometacarpal joint replacement surgery, websites requiring logins, YouTube videos, and websites composed solely of videos were excluded from this analysis. Medical journals were also excluded in concordance with previous studies that found the complexity of medical journals beyond the understanding of the general population.^14^, ^15^, ^16^ This methodology is concordant with similar studies previously published in the literature.^14^, ^15^, ^16^^,^^25^^,^^26^^,^^28^ Fig. 1 illustrates a breakdown of the methodology.

Websites chosen for analysis were further categorised according to methodologies established in previously published studies.^29^, ^30^, ^31^ Categories include (1) "commercial" websites referring to websites affiliated with commercial organizations that act as sources of health information; (2) "academic" websites referring to websites affiliated with universities, academic hospitals, and academic societies; (3) "non-academic" websites referring to websites associated local hospitals and private practices; (4) "non-profit" websites referring to those maintained by a national government or government organizations or a non-for-profit organization; and (5) "miscellaneous" websites referring to websites that do not fit the aforementioned four categories.

### 2.2 Analysis of readability

Websites were uploaded onto an online readability software (WEB FX).^32^ Website URLs such as pdf links that failed to be input directly into the online tool were reformatted to remove non-narrative text (eg. headings, logos, descriptors that did not form full sentences) before being imported into the text analyser. Readability was assessed using two validated algorithms for readability which determined readability according to the number of characters or syllables per word: (1) the Flesch Reading Ease Score (FRES) and (2) the Flesch-Kincaid Reading Grade Level (FKGL).^33^^,^^34^ These scores are summarised in Table 2. A more detailed breakdown of the FRES score can be found in Table 3. In addition to online healthcare-related resources needing to be written at or below a US sixth-grade level,^13^, ^14^, ^15^, ^16^ previous studies also determined that a FRES score of at least 65 is required for the material to be acceptable for public reading standards.^14^, ^15^, ^16^

**Table 2:** Summary of readability scores.

Score	Scoring basis	Formula
Flesch Reading Ease Score (FRES)	Index score from 0 to 100.	(206.835 ​− ​(84.6 ​× ​ASW) ​− ​(1.015 ​× ​ASL)
Flesch-Kincaid Reading Grade Level (FKGL)	Grade level.	(11.8 ​× ​ASW) ​+ ​(0.39 ​× ​ASL) −15.59

Abbreviations: ASL ​= ​number words/number sentences; ASW ​= ​number syllables divided by number of words.

**Table 3:** Summary of the flesch reading ease score.

Score	Level of education	Description
90–100	5th Grade	Very easy to read. Easily understood by an average 11-year-old student.
80–90	6th Grade	Easy to read. Conversational English for consumers.
70–80	7th Grade	Fairly easy to read.
60–70	8th to 9th Grade	Plain English. Easily understood by 13-year to 15-year-old students.
50–60	10th to 12th Grade	Fairly difficult to read.
30–50	College	Difficult to read.
0–30	College Graduate	Very difficult to read. Best understood by university graduates.

### 2.3 Analysis of quality

The Patient Education Materials Assessment Tool for Printable materials (PEMAT) was used to evaluate understandability, the ability of users to process and explain key messages of the resource, and actionability, the ability of users to identify action steps or advice for the benefit of their own health based on information provided.^35^ The PEMAT includes 17 items for understandability and 7 items for actionability.^35^ Item were scored as yes (score ​= ​1) or no (score ​= ​0) and then summed and divided by the total number of items to produce separate scores for understandability and actionability. Scores were expressed as a percentage, 0 ​% meaning not understandable or actionable at all, 100 ​% meaning easily understood or actionable, and a score of below 70 ​% being considered poorly understandable and poorly actionable. The use of the PEMAT to measure website understandability and actionability is concordant with previously conducted studies published in the literature.^36^, ^37^, ^38^

The quality of websites was also evaluated using a modified DISCERN tool consisting of 16 questions regarding bias, risks and benefits, relevance, and overall quality of information.^39^^,^^40^ While the traditional DISCERN tool has answer choices ranging from 1 to 5 (1 ​= ​no; 2/3 ​= ​partially; 4/5 ​= ​yes),^40^ this modified tool increases precision by instead ranging answers from 1 to 3 (1 ​= ​no; 2 ​= ​partially; 3 ​= ​yes.^29^ Total DISCERN scores ranged from 16 to 48, with higher scores representing higher quality, and grouped into categories of Excellent (40–48), Good (34–39), Fair (28–33), Poor (22–27), and Very Poor (16–21). This method of qualitative assessment is similar to other studies previously published in the literature.^29^, ^41^, ^42^

### 2.4 Statistical analysis

Statistical analysis was performed using the GNU PSPP V2.0.0 statistical software. P values ​< ​0.05 were deemed significant. Analysis of variance (ANOVA) testing was performed to determine the difference between categories. One-sample t-tests were used to compare FRES scores with the recommended standard of 65, while grade-level scores were compared with the recommended sixth-grade standard. Pearson correlation coefficient testing was carried out to determine whether there was any association between FRES readability scores and PEMAT scores, and between FRES readability scores and DISCERN scores.

### Ethical approval

Patients and the public were not involved in this study. This internet-based study without human subjects did not require institutional review board approval.

## 3 Results

A total of 34 websites underwent qualitative analysis. A list of these websites can be found in the Appendix. Among these, there were 5 commercial websites, 3 academic websites, 24 non-academic websites, 1 non-profit website, and 1 miscellaneous website. Readability scores by FRES and FKGL, PEMAT Understandability, PEMAT Actionability, and DISCERN for all websites were analysed and are shown in Table 4. Mean FRES, FKGL, PEMAT Understandability, PEMAT Actionability, and DISCERN scores are further subdivided by website type and shown in Table 5.

**Table 4:** Summary of descriptive statistics.

Score	FRES	FKGL	PEMAT Understandability	PEMAT Actionability	Total DISCERN
N (Valid)	34	34	34	34	34
N (Missing)	0	0	0	0	0
Mean	54.60	8.19	76.82	36.18	32.00
Median	57.15	7.75	76.69	40.00	32.00
Mode	53.20	7.70	69.23	60.00	27.00
SD	7.91	1.80	9.43	24.12	4.29
Skewness	−1.00	0.80	0.37	−0.50	0.37
SE of skewness	0.40	0.40	0.40	0.40	0.40

Abbreviations: FKGL = Flesch-Kincaid Reading Grade Level; FRES = Flesch Reading Ease Score; SE ​= ​standard error; PEMAT = Patient Education Materials Assessment Tool.

**Table 5:** Summary of descriptive statistics.

Website type (n)	Mean FRES	Mean FKGL	Mean PEMAT Understandability	Mean PEMAT Actionability	Mean Total Discern
Commercial (5)	52.34	7.68	76.11	46	35.8
Academic (3)	59.33	8.57	89.58	60	31
Non-academic (24)	54.49	8.31	76.32	34.17	31
Non-profit (1)	55.7	7.2	61.54	0	35
Miscellaneous (1)	53.2	7.7	69.23	0	37

### 3.1 Readability of websites

**Fig. 2:**
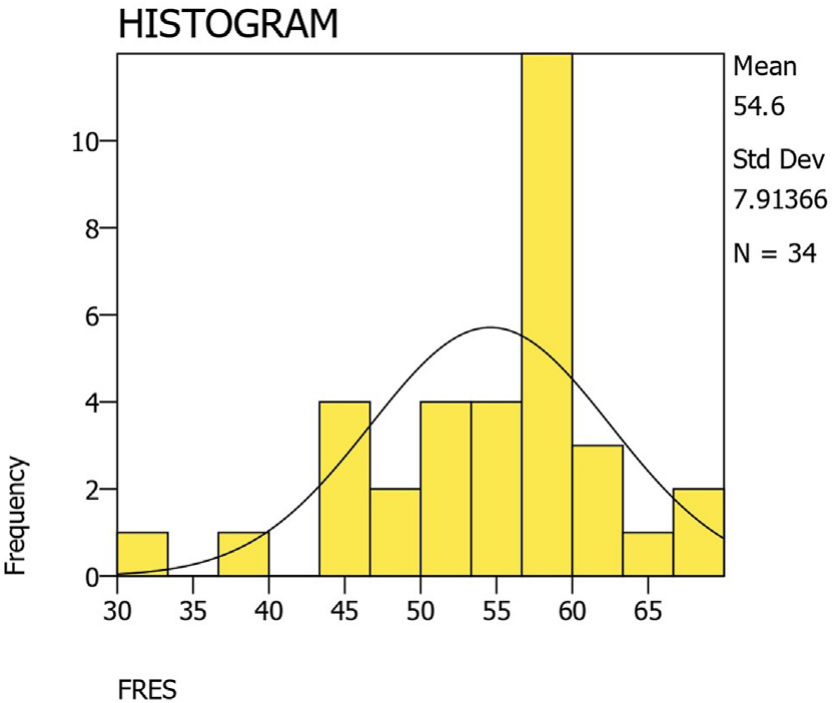
Histogram of flesch reading ease scores (FRES).

The average FRES score was 54.60 ​± ​7.91 (range 30.30–67.80), placing the data readability at a 10th to twelfth-grade level and “fairly difficult to read”. One-sample t-testing against the recommended FRES score of 65 showed that FRES scores were significantly higher than the acceptable standard (p ​< ​0.001, CI −13.16 to −7.64). Only 3 (8.82 ​%) websites had a FRES score >65. 8 websites (23.53 ​%) had a FRES score <50, implying that at least a college-level education was needed to read the material. A one-way ANOVA test showed no significant difference between FRES scores based on categories (p ​= ​0.839). FRES scores are illustrated in Fig. 2.

**Fig. 3:**
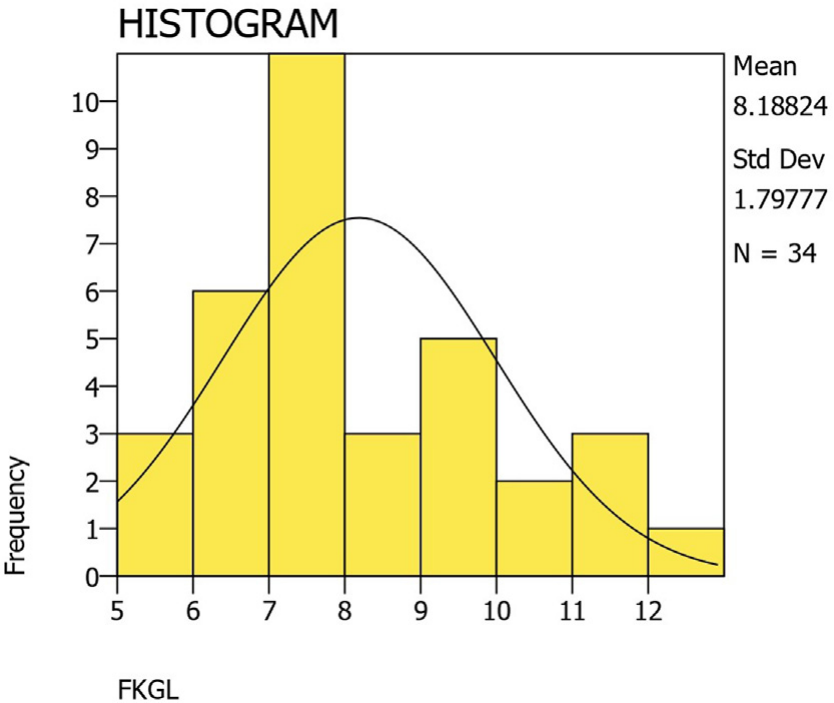
Histogram of flesch-kincaid reading grade levels (FKGL).

The average FKGL score was 8.19 ​± ​1.80 (range 5.60–12.90). One-sample t-testing against the recommended sixth grade standard showed that FKGL scores were significantly higher than the acceptable standard (p ​< ​0.001, CI 1.56 to 2.82). Only 3 (8.82 ​%) websites were written at or below a sixth-grade level according to the FKGL score. A one-way ANOVA test showed no statistically significant difference between categories (p ​= ​0.919). FKGL scores are illustrated in Fig. 3.

### 3.2 Quality of websites

The average PEMAT percentage score for understandability was 76.82 ​± ​9.43 (range 61.54–93.75). The average PEMAT percentage score for actionability was 36.18 ​± ​24.12 (range 0.00–60.00). One-sample t-testing against the recommended score of >70 ​% showed that PEMAT scores for understandability (p ​< ​0.001, CI 3.53 to 10.11) and actionability (p ​< ​0.001, CI -42.24 to −25.41) were significantly higher than the acceptable standard. Although 22 (64.71 ​%) of websites met the acceptable standard of 70 ​% for understandability, none of the websites met the acceptable standard of 70 ​% for actionability.

The average total DISCERN score was 32.00 ​± ​4.29 (range 24.00–42.00). A one-way ANOVA test showed no statistically significant difference between categories (p ​= ​0.115). The average total DISCERN scores of academic and non-academic websites were both Fair, scoring at 31.00 ​± ​4.36 (range 26.00–34.00) and 31.00 ​± ​3.75 (range 24.00–42.00) respectively. The average total DISCERN scores of commercial, non-profit, and miscellaneous websites were Good, scoring 35.80 ​± ​5.26 (range 27.00–41.00), 35, and 37 respectively.

### 3.3 Correlation between readability and quality

There was no significant correlation FRES scores and PEMAT understandability scores (r ​= ​0.097, p ​= ​0.587). However, there was a statistically significant correlation between FRES scores and PEMAT actionability scores (r ​= ​0.380, p ​= ​0.027), and between FRES scores and DISCERN scores (r ​= ​−0.353, p ​= ​0.044).

## 4 Discussion

This study is the first to consider the readability of the information patients will have access to on the internet for thumb CMC joint replacements. It used six different tests to determine readability. However, this study found that for online resources regarding thumb CMC joint replacement, only 5.56–16.67 ​% of websites analysed were readable at or below a sixth-grade level while 12 (33.33 ​%) needed at least a college-level education to be read. Healthcare websites need to provide reliable, timely, and high-quality information at a level which is considered easily comprehensible by patients and caregivers.^15^^,^^43^ Studies show that patient satisfaction also has a direct correlation with patient comprehension of information such as disease processes, treatment options, and expectations.^44^

In orthopaedic surgery, Daraz et al. showed a mean readability grade level of grade 10 to 15 among orthopaedic surgery-related websites.^9^ In the field of hand and wrist surgery, Cook et al. found that among 42 institutions with plastic and orthopaedic surgery training programs, patient education materials regarding hand surgery were written at an 11.92-grade reading level with information regarding de Quervain's tenosynovitis having the highest grade level for plastic surgery procedures (13.45) and hand arthritis having the highest grade level for orthopaedic surgery procedures (12.82).^44^ Foster et al. found that among 170 online patient resources provided by the American Society for Surgery of the Hand (ASSH), the American Association for Hand Surgery (AAHS), and the American Academy of Orthopaedic Surgeons (AAOS), the mean FRES score was 57.3 ​± ​7.7 and the mean FKGL score was 9.1 ​± ​1.3.^45^ Furthermore, studies analysing online hand surgery patient educational materials published by the ASSH showed an average FKGL score of 10.4 in 2008,^46^ 8.5 in 2015,^47^ and 9.8 in 2020.^48^ This trend suggests that despite the improvement observed between 2008 and 2015, materials published between 2015 and 2020 were being written above recommended reading levels.^48^

The availability of online medical resources being written above recommended reading levels is also prevalent in other fields of medicine and surgery.^12^, ^13^, ^14^, ^15^, ^16^^,^^49^ A systematic review of 157 cross-sectional studies evaluating 7891 websites demonstrated that the mean readability grade level across websites ranged from grade 10 to 15.^9^ Shukla et al. found that among 40 websites related to uterine artery embolization, none were written at or below a sixth-grade level, averaging a FRES score of 43.98.^12^ Vargas et al. found that among 80 websites related to liposuction, all exceeded the target sixth-grade level and had an overall 13.6-grade reading level (range 10–16 grade) and average FRES score of 36.^50^ A comparison of studies of websites related to Dupuytren's contracture by Santos et al. carried out in 2016 and Hosseinzadeh et al. carried out in 2021 showed a worsening in readability levels across the FRES and FKGL scores.^13^^,^^49^ Hosseinzadeh et al. also found that among 73 websites, none were written at or below a sixth-grade level, averaging a FRES score of 48.6 ​± ​8.0 (31.7–68.2).^13^ Therefore, while websites that discuss thumb CMC joint replacements may not meet recommended standards, they may be more accessible to the public than online resources for other medical conditions or treatments.

The quality assessment using the DISCERN tool found that 6 websites were “Poor”, 15 were “Fair”, 11 were “Good”, and 2 were “Excellent”. The average score “Fair”. Websites generated by the search were found to mostly be based on expert opinions or patient stories with few actually citing sources of information. Although most websites described what a thumb joint replacement surgery entails, some did not clearly state the benefits and risks of the procedure, or make alternative options known to readers. The mean PEMAT score of 76.82 ​% for understandability was higher than the mean PEMAT score of 36.18 ​% for actionability. This trend of PEMAT scores for understandability exceeding PEMAT scores for actionability is observed in other analyses of online patient resources, ranging from websites dedicated to rotator cuff repairs (64.6 ​% versus 29.5 ​%),^51^ brain arteriovenous malformations (86.6 ​% versus 54.6 ​%),^52^ and COVID-19 (62.1 ​% versus 37.9 ​%).^53^ In our study, majority of websites met the 70 ​% cutoff for understandability while none met the cutoff for actionability. Our results suggest that although average scores are considered understandable, they were all poorly actionable, showing that patients may be provided with information that is understandable but not given clear instructions to act on regarding pre-operative or post-operative steps they should undertake to maximise positive outcomes. Websites that scored poorly for understandability usually did not utilise clear subheadings, did not explain medical terms or statistics used, or scattered images around the webpage without titles or captions. Actionability scores were mostly poor because some websites did not clearly identify at least one pre-operative or post-operative action readers could take while websites that did identify actions either failed to address readers directly or failed to break down instructions into manageable, explicit steps.

To improve readability and understandability, the personnel involved in the writing of online resources should ideally assume readers have difficulty understanding the information and write materials in a comprehensible way by using simple words, avoiding jargon, and supplementing text with videos and diagrams.^15^ Visual aids should be specifically selected to reinforce the material rather than clutter the webpage. Written text should also be evaluated using readability software before being published online.^16^ Patient input and preferences are also important to account for when creating educational materials that are comprehensible and of high quality.^13^ To improve the quality of online resources, sources of information should be kept up-to-date and clearly stated on websites while details of the procedure, benefits, risks, and alternatives should also be made available on the webpage to reduce bias. To improve actionability, especially regarding pre-operative and post-operative instructions, webpages should address readers directly, use an “active” voice, and break down actions into manageable steps for readers to act on more easily.

This study was not without limitations. Regarding limitations of the search, the first two pages of results were screened which may have resulted in relevant websites on subsequent pages being excluded. Materials on the internet also change from day to day and the top 20 search results may differ depending on a user's cookies or location. Regarding limitations of the analysis, the online software tool does not account for illustrations and videos that may aid in patient understanding. Tools used to assess readability were not originally designed to assess health literature and do not use health content in their validation.^54^ Algorithms used also determine readability according to the number of characters or syllables per word without considering the meaning of each word. This may result in an inaccurate representation of how difficult a word is to understand. There are also more readability and comprehension instruments than the two utilized in this review, such as the Dale-Chall Formula, the Gunning-Fog Index, the Fry Readability Graph, and the Simple Measure of Gobbledygook (SMOG).^55^ Furthermore, the World Health Organization-endorsed Health On The Net Foundation code (HONcode) which was previously used to verify a website's ethics based on authority, complementarity, confidentiality, attribution, justifiability, transparency, financial disclosure, and advertising policy was discontinued on 15 December 2022. Therefore, although this tool was used by previous publications analysing the readability and quality of online resources, we relied instead on the PEMAT and DISCERN tools to assess the quality of websites.

## 5 Conclusion

Current online resources regarding thumb CMC joint replacements are written above the recommended reading grade level. Poor patient understanding of the available online resources may lead to negative outcomes. As such, providers of these resources need to ensure that materials are accessible to the average reader, regardless of whether they are academic websites or private healthcare websites. This can be ensured by using simple words, avoiding jargon, and analysing texts with readability software before publishing the materials online. Regarding the quality of websites, visual aids and tables may be useful in providing information in a more understandable way for patients while clear stepwise pre-operative and post-operative instructions will increase the quality and usefulness of the information provided.

## Funding

This research did not receive any specific grant from funding agencies in the public, commercial, or not-for-profit sectors.

## Declaration of competing interest

The authors declare that they have no known competing financial interests or personal relationships that could have appeared to influence the work reported in this paper.
