# Supplementary material for: A cross-sectional quantitative analysis of the readability and quality of online resources regarding thumb carpometacarpal joint replacement surgery
Source: J Hand Microsurg. 2024 Jun 26;16(4):100119. doi: 10.1016/j.jham.2024.100119 (PMC11369735; doi:10.1016/j.jham.2024.100119)
Supplement: Multimedia component 1 [file mmc1.docx]

**Appendix Legend**

Appendix A: Included websites from each search engine.

**Appendix A**

| Search engine | Included websites |
| --- | --- |
| Google | https://www.stgeorgesurgical.com/procedure/thumb-arthroplasty/  https://www.arthritis.org/health-wellness/treatment/joint-surgery/preplanning/five-surgeries-for-thumb-arthritis  https://charmssingapore.com/treatment_surgery/basal-thumb-joint-replacement/  https://www.prolianceorthopedicassociates.com/thumb-arthroplasty  https://www.medicalnewstoday.com/articles/thumb-arthritis-surgery  https://www.topdoctors.co.uk/medical-articles/exploring-thumb-joint-replacement-a-comprehensive-overview  https://hje.org.uk/treatments/thumb-joint-replacement/  https://www.pugetsoundorthopaedics.com/specialties/joint-replacement/thumb-arthroplasty/  https://www.arspinesportstherapy.com/Injuries-Conditions/Hand/Surgery/Artificial-Joint-Replacement-of-the-Thumb-Spine-and-Sports-Guide/a~3917/article.html  https://www.circlehealthgroup.co.uk/health-matters/patient-stories/jan-zakary-cmc-joint-replacement-surgery  https://www.ddchirurgiedelamain.ca/procedure/thumb-arthroplasty/  https://www.singhealth.com.sg/patient-care/conditions-treatments/CMC-Arthroplasty  https://minnesotavalleysurgerycenter.com/hand-surgery/when-can-i-return-to-work-following-a-thumb-cmc-arthroplasty/  https://orthop.washington.edu/sites/default/files/hand-center/Post-Surgery-Thumb-CMC-July-2013.pdf  https://msapc.com/hand-center/aftercare/thumb-joint-arthroplasty/  https://www.verywellhealth.com/carpometacarpal-joint-arthroplasty-5208414  https://www.healthline.com/health/carpal-meta-carpal-cmc-arthroplasty  https://sussexhandsurgery.co.uk/downloads/surgery/hand/Thumb%20CMC%20Joint%20Replacement.pdf  https://www.circlehealthgroup.co.uk/health-matters/patient-stories/sandra-taylor-cmc-joint-replacement-surgery  https://minnesotavalleysurgerycenter.com/hand-surgery/treatment-for-thumb-arthritis-when-to-consider-carpometacarpal-cmc-arthroplasty/  https://www.bristolhandsurgery.com/thumb-cmc-replacement-surgery/  https://www.swhmicro.com/contents/thumb-cmc-basal-joint-arthroplasty-thumb-joint-reconstruction  https://www.concordortho.com/patient-resources/patient-education/topic/4dd3b8e730609bbcf468aed4fc0ea7a5  https://www.drelzaim.com/thumb-cmc-joint-arthroplasty-haissam-s-elzaim-orthopaedic-surgeon-mcallen.html  http://myplasticsurgeon.com/hand-surgery/osteoarthritis-base-of-thumb  https://www.lmh.org/get-care/orthokansas/hand-wrist/carpal-meta-carpal-cmc-arthroplasty/  https://minnesotavalleysurgerycenter.com/hand-surgery/technology-with-thumb-arthritis-can-cmc-arthroplasty-make-texting-pain-free/ |
| Bing | https://www.hoagorthopedicinstitute.com/blog/2019/july/thumb-joint-replacement-surgery-purpose-procedur/  https://www.healthline.com/health/hand-surgery-for-arthritis  https://www.healthline.com/health/carpometacarpal-arthroplasty-techniques  https://www.martinortho.care/contents/patient-education/thumb-cmc-arthroplasty  https://www.drugs.com/cg/thumb-arthroplasty.html  https://www.sgh.com.sg/patient-care/specialties-services/Hand-Surgery/Documents/1st-CMCJ-arthroplasty.pdf  https://tcomn.com/wp-content/uploads/2016/06/Basal-Thumb-Joint-CMC-Arthroplasty-Surgery-1.pdf |
